# Enhanced Cellular Uptake of H-Chain Human Ferritin Containing Gold Nanoparticles

**DOI:** 10.3390/pharmaceutics13111966

**Published:** 2021-11-19

**Authors:** Italo Moglia, Margarita Santiago, Simon Guerrero, Mónica Soler, Alvaro Olivera-Nappa, Marcelo J. Kogan

**Affiliations:** 1 Department of Pharmacological and Toxicological Chemistry, Faculty of Chemical and Pharmaceutical Sciences, University of Chile, Santiago 8380494, Chile; italo.moglia@gmail.com; 2 ACCDiS—Advanced Center for Chronic Diseases, Faculty of Chemical and Pharmaceutical Sciences, University of Chile, Santiago 8380494, Chile; simon.daiblogt@gmail.com; 3 CeBiB—Center for Biotechnology and Bioengineering, Faculty of Physical and Mathematical Sciences, University of Chile, Santiago 8370456, Chile; santiago.margarita@gmail.com; 4 Interdisciplinary Research Institute in Biomedical Science—I3CBSEK, SEK University, Santiago 7520317, Chile; 5 Department of Chemical Engineering, Biotechnology and Materials, Faculty of Physical and Mathematical Sciences, University of Chile, Santiago 8380494, Chile; msoler@ing.uchile.cl

**Keywords:** ferritin, gold nanoparticles, cellular internalization, cellular viability, theranostics

## Abstract

Gold nanoparticles (AuNP) capped with biocompatible layers have functional optical, chemical, and biological properties as theranostic agents in biomedicine. The ferritin protein containing in situ synthesized AuNPs has been successfully used as an effective and completely biocompatible nanocarrier for AuNPs in human cell lines and animal experiments in vivo. Ferritin can be uptaken by different cell types through receptor-mediated endocytosis. Despite these advantages, few efforts have been made to evaluate the toxicity and cellular internalization of AuNP-containing ferritin nanocages. In this work, we study the potential of human heavy-chain (H) and light-chain (L) ferritin homopolymers as nanoreactors to synthesize AuNPs and their cytotoxicity and cellular uptake in different cell lines. The results show very low toxicity of ferritin-encapsulated AuNPs on different human cell lines and demonstrate that efficient cellular ferritin uptake depends on the specific H or L protein chains forming the ferritin protein cage and the presence or absence of metallic cargo. Cargo-devoid apoferritin is poorly internalized in all cell lines, and the highest ferritin uptake was achieved with AuNP-loaded H-ferritin homopolymers in transferrin-receptor-rich cell lines, showing more than seven times more uptake than apoferritin.

## 1. Introduction

Gold nanoparticles (AuNP) have great potential as theranostic agents in biomedicine due to their optical, chemical, and biological properties, such as surface plasmon resonance, low chemical reactivity, and reduced cell toxicity. The use of AuNP for cell targeting requires a protective layer that provides biocompatibility and prevents nonspecific adsorption of proteins as protein coronae, which could impart new biological properties impacting nanoparticle reactivity, bioavailability, and pharmacokinetics, and ultimately lead to cytotoxicity or immunotoxicity [1]. Ideally, the protective biocompatible layer must improve circulating half-life and minimize the reticuloendothelial system uptake that rapidly removes nanoparticles from the circulatory system to the liver, spleen, or bone marrow [2].

Different molecules have been evaluated as protective layers or coating agents for AuNPs. For example, polymers such as polyethylene glycol [3], biomolecules such as cysteine [4,5], peptides such as glutathione [6,7], and synthetic peptides [8] and proteins such as bovine serum albumin (BSA) [9,10,11,12,13] and ferritin [14,15,16,17,18] have been used in different biomedical applications. 

The use of ferritin (FT) meets many desirable characteristics for a good coating agent for AuNPs in nanomedical applications. This is mainly due to ferritin’s particular quaternary structure, which comprises a tissue-specific mix of 24 subunits of heavy (H) and light (L) chain ferritins, forming a hollow sphere protein complex that can be used as a container for metallic nanoparticles. Both subunit types (H or L) have similar amino acid substitutions that confer functions specific to each subunit [19,20]. Ferritin is completely biocompatible because it is endogenous to humans; its protein shell provides a highly stable protective layer for inorganic nanoparticle coating [17,21,22,23,24,25,26], and its cavity has been successfully used as a nanoreactor to prepare monodisperse metallic nanoparticles [18,22,27]. The synthesis of AuNP in ferritins from different origins has been reported using horse spleen FT [14,15,16], mutant L-chain horse spleen FT [28], H-chain human FT [17,29], mutant H-chain human FT [30], and *Archaeoglobus fulgidus* FT [26].

Another relevant feature of ferritin for nanoparticle targeting is that it can be uptaken by different cell types and tissues through a receptor-mediated endocytosis process. The primary receptor for ferritin is transferrin receptor-1 (TfR1), expressed in many cell lines [31] and tumor tissues [32,33]. Furthermore, two other known receptors interact with ferritin: the Tim2 receptor, expressed on oligodendrocytes [34] and B-cells and in the liver and kidney [35,36], and the Scara-5 receptor, expressed in some specific embryonic kidney cell types (stromal and capsular cells, [37]), the MCF-7 breast cancer cell line [38], and blood-retinal barrier cells [39]. So far, these three ferritin receptors are known to recognize the ferritin H-subunit, except for Scara5, which can also interact with the L-subunit of ferritin [40]. Additionally, ferritin can be genetically modified or chemically conjugated to add cell specificity for different receptors to further enhance specificity or target more than one possible receptor or cell type.

Promising results in biomedical imaging in mice have been observed after tail vein injection of horse spleen ferritin and human H-chain ferritin homopolymer containing NIR fluorescent gold nanoclusters, which acted as excellent fluorescent probes for whole-body imaging, with particular targeting to the kidneys [16,17]. Additionally, horse spleen ferritin containing AuNP targeted to the MCF-7 cancer cell line (by chemical conjugation with 2-amino-2-deoxy-glucose) has shown increased computer tomography contrast enhancement [41]. However, despite these few efforts to evaluate the use of gold nanostructures in ferritin for biomedical imaging, the use of human H and L ferritin homopolymers for gold nanoparticle synthesis and the evaluation of their toxicity and internalization in different cell lines are still underexplored aspects that must be addressed.

Considering the potential biomedical use of ferritin as a theranostic agent in humans, we tested both human homopolymers (H and L) as nanoreactors to synthesize gold nanoparticles. We characterized each FT-AuNP homopolymer and analyzed their cytotoxic effect and ability to be taken up by different cell lines. The results show how the intrinsic characteristics of each homopolymer influence the successful synthesis of AuNPs and reveal that the metallic content provides advantageous properties to the FT-based theranostic agent.

## 2. Materials and Methods

### 2.1. Plasmid Construction

cDNA from colorectal adenocarcinoma (Caco-2) cells, donated by Dr. Tulio Nuñez, was used for the amplification of H-chain ferritin (FTH) and L-chain ferritin (FTL) cDNAs with primers designed based on NCBI reference sequences (NM_002032.3 and NM_000146.4). For subsequent cloning into the bacterial expression plasmid pET-22b, the cDNAs were amplified with primer pairs FTH-F/FTH-R and FTL-F/FTL-R (Table 1), which introduced restriction endonuclease cleavage sites for NdeI and XhoI into the 5′ and 3′ regions, respectively. The sequences of constructed plasmids pET-28a-FTH and pET-28a-FTL were verified by DNA sequencing.

### 2.2. Ferritin Expression and Purification

Plasmids were transformed into *Escherichia coli* BL21 DE3, and protein expression was induced by growing the cells in autoinduction media [42] for 20 h at 25 °C, with shaking at 250 rpm. Cells were harvested at 6000× *g* for 10 min, resuspended in 50 mM Tris pH 8, and disrupted by sonication. Clarified supernatants were heated at 75 °C for 10 min, cooled on ice for 5 min, and denatured protein contaminants were removed by centrifugation. Then, ferritin extracts were purified through a Q-sepharose column eluting with a linear NaCl gradient (0–1 M) using an ÄKTA Avant system. Ferritin-containing fractions were concentrated and finally purified using a Superdex 200 Increase 10/300 GL column, selecting monomer protein elution for further AuNP synthesis.

### 2.3. Gold Nanoparticle Synthesis in Apoferritin

Gold nanoparticle (AuNP) synthesis was performed following the protocol published by Fan 2010 [15] with slight modifications. Briefly, 13.3 μL of a 100 mM solution of HAuCl_4_ were added to 1 mL of 2 mg/mL ferritin solution in Tris 50 mM pH 8 (equivalent to 300 Au atoms per ferritin, 300 Au/FT), and the solution was incubated for 2 h at 4 °C. Afterward, excess metallic salts were removed by dialysis against Tris 50 mM pH 8. A first chemical reduction was performed by adding 13.3 μL of a 100 mM solution of NaBH_4_ and incubating at 4 °C overnight. The next day, another 13.3 μL fraction of 100 mM HAuCl_4_ (300 Au/FT) was added to the ferritin solution and incubated for 2 h at 4 °C. Excess auric salts were removed by dialysis against Tris 50 mM pH 8. A second chemical reduction was performed by adding 13.3 μL of a 100 mM ascorbic acid solution and incubating at 4 °C overnight. Samples were centrifuged at 14,000 rpm for 10 min at 4 °C. Finally, the soluble fraction was dialyzed against Tris 50 mM pH 8 and stored at 4 °C. Each gold nanoparticle synthesis experiment in ferritin was performed in duplicate.

### 2.4. Characterization of Ferritin AuNP

Protein quantification was carried out using the modified Lowry method (Thermo Fisher), with BSA as a reference. Optical properties of protein samples with nanoparticles were measured using UV–vis spectroscopy in a Lambda 25 spectrophotometer (Perkin Elmer, Waltham, MA, USA). Ferritin samples containing AuNPs were subjected to size exclusion chromatography (SEC) using a Superdex 200 Increase 10/300 GL in an FPLC Äkta Avant instrument (GE Healthcare Life Sciences), measuring absorbance at 280 (A_280 nm_) and 550 nm (A_550 nm_) for protein and AuNP detection, respectively. Dynamic light scattering (DLS) was used to measure the colloidal size distribution and Z-potential of samples using a Zetasizer Nano instrument (Malvern). The structural integrity of the samples was also characterized by native polyacrylamide gel electrophoresis (PAGE). TEM characterization was performed using a JEM-1010 JEOL microscope operated at 100 KV from the University of Barcelona. The statistical analysis of gold nanoparticle size distribution was performed using ImageJ software by sampling over 400 particles.

### 2.5. Cell Culture and Incubation with Ferritin AuNP

Human colon adenocarcinoma HT29 cells (ATCC), human embryonic kidney HEK293 cells (ATCC), mouse brain cortex BEND3 cells (ATCC), and mouse embryonic fibroblasts 3HC/10T1/2 cells (ATCC) were maintained in Dulbecco’s modified Eagle’s medium (DMEM HG medium) (Gibco™; Thermo Fisher Scientific, Waltham, MA, USA) supplemented with 10% fetal bovine serum (FBS; Bioind) and antibiotics (penicillin 100 UI/mL, streptomycin 100 μg/mL), at 37 °C in 5% CO_2_. For uptake assays, cells were seeded in a 96-well plate at a density of 2500 cells/100 µL of medium and incubated for 24 h with FTH and FTL containing gold nanoparticles or iron oxides (as loading control). ApoFTH and ApoFTL were used as control. The added ferritin samples never exceed 10% of cell culture volume.

### 2.6. Viability Assays

In order to evaluate cell viability after incubation with ferritin samples containing AuNPs, the medium was replaced with fresh culture medium containing 10% of tetrazolium compound in an MTS^®^ assay (CellTiter 96^®^ Aqueous Non-Radioactive Cell Proliferation Assay), according to the manufacturer’s instructions (Promega, Madison, WI, USA). The soluble formazan produced by live cells was detected by absorbance at 490 nm on a multiscan reader (Synergy-H4, Biotek, VT, USA). Background values contributed by excess cell debris and bubbles obtained by measuring at 650 nm were subtracted.

### 2.7. Fixation and Confocal Microscopy

Cells were fixed with 4% paraformaldehyde (PFA) at 4 °C for 10 min. Then, residual PFA was blocked with 50mM glycine. Then, cell membranes were permeated with 0.1% Triton X-100 for 10 min and blocked with 2% BSA for 30 min. Next, cells were incubated with a dilution of 1:100 of anti FTL (PA5-29599, Thermo Fisher Scientific, Bedford, MA, USA) or anti FTH (sc-376594, Santa Cruz Biotechnology Inc, Dallas, TX, USA) in 2% BSA for 1 h at 37 °C. Then, antibodies were rinsed with 2% BSA and incubated with 1:300 secondary antibodies (Anti Rabbit Alexa488, ^®^Invitrogen or Anti Mouse Alexa546, ^®^Molecular Probes, Salem, OR, USA). After several rinses, cells were incubated with a 1:2000 DAPI dilution. The cell-containing coverslips were mounted on glass slides using Fluoromount G (Thermo Fisher Scientific, Bedford, MA, USA) for imaging and analysis in a Zeiss confocal microscope. Images were taken using a 63X aqueous lens with glycerol-based immersion medium (IR: 1.4) employing a digital zoom of 0.5X by plane and 1X for z stacking. Ten different fields of the glass slide with cell homogeneity were measured and quantified using ImageJ software. Cell fluorescence was measured in the region of interest and corrected by integrated density adjusted by mean background fluorescence in the region of interest of the cell area. Mean fluorescence intensity (ROI of Cell Fluorescence Corrected = Integrated Density of ROI (Area of ROI x Mean Fluorescence of Background Readings) of treated cells with metal-containing ferritin was divided by the mean fluorescence of the apoferritin control group and, finally, was registered, plotted and analyzed using GraphPad software.

## 3. Results and Discussion

### 3.1. Gold Nanoparticles Synthesis and Characterization

**Figure 1:**
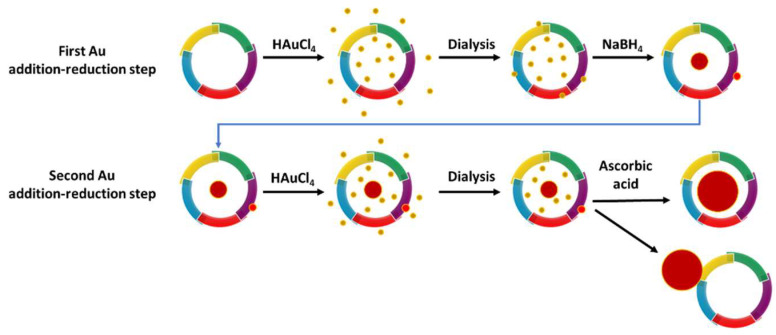
Diagram of the 2-step gold nanoparticle synthesis protocol in recombinant human ferritin. It cannot be discarded that some gold atoms could be on the outer ferritin surface after the first dialysis, giving rise to nanoparticles outside the formation.

Recombinant human H-ferritin homopolymer (FTH) and L-ferritin homopolymer (FTL) were used as nanocontainers for the synthesis of AuNPs to obtain FTH-AuNP and FTL-AuNP, respectively, following a protocol with two successive gold addition–reduction steps based in a previous protocol published by Fan 2010 [15] (Figure 1). Potentially, gold nanoparticle growth could occur in the outer or inner surface of the ferritin shell.

**Figure 2:**
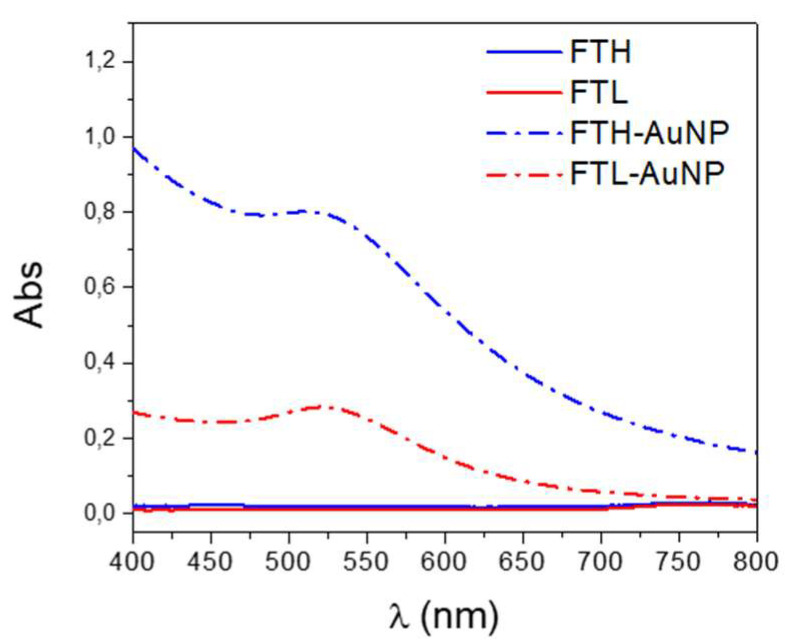
Absorbance spectra of FT-AuNP samples. Gold-containing samples (FTH-Au NP and FTL-Au NP) are represented by dash-and-dot lines to compare them to the apo-forms of FTH and FTL shown as continuous lines.

Ferritin-containing gold nanoparticles (FT-AuNPs) were characterized by UV-vis spectroscopy in the range of 400 to 800 nm to evaluate the characteristic surface resonance plasmon (SRP) absorbance peak of the AuNPs [43] (Figure 2). The spectra of FT-AuNP samples exhibited the SRP absorbance peak at 511 and 520 nm in FTH and FTL, respectively. The slight shift observed between both AuNP-ferritin SRP peaks and the different absorbance intensities between FTH and FTL reflect not only differences in the metallic core size distribution of the gold nanoparticles [44] but also the effect of the dielectric constant of the nanoparticle environment in each homopolymer, which is different due to the specific amino acid composition interacting with the AuNP surface.

**Figure 3:**
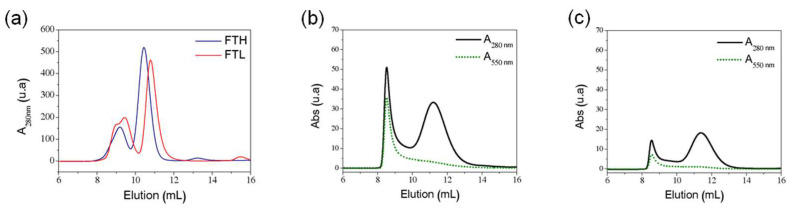
Size exclusion chromatography of FTL and FTH samples. Elution profile of (**a**) ApoFTH and ApoFTL, (**b**) FTH-AuNP and (**c**) FTL-AuNP.

The FT-AuNP samples were further characterized by studying their structural integrity using size exclusion chromatography (SEC) and dynamic light scattering (DLS). Absorbance at 280 nm (A_280nm_) was used in chromatographic analysis to detect the protein and the absorbance at 550 nm (A_550nm_) for AuNP detection. The chromatographic characterization of apoferritin homopolymer samples at 280 nm exhibited two prominent elution peaks for each sample, with a slight difference in elution volumes. The ApoFTH profile showed a first elution peak at 9.1 mL and a second peak at 10.4 mL. ApoFTL exhibits a broad first protein elution with two overlapping peaks at 8.9 and 9.4 mL and a second elution peak at 10.8 mL (Figure 3A). These elution profiles indicate two different subpopulations of ferritin in the original sample that have also been reported by other authors [22,45,46,47]. The first protein elution peak with a higher molecular weight corresponds to ferritin cage oligomers (dimers or trimers), while the second elution peak contains ferritin cage monomers, corroborated by native PAGE analysis (Figure S1). In a previous work of our group, horse spleen ferritin (HSF) showed the same elution profile and hydrodynamic diameter measurement in the SEC collected fractions, with a first elution peak having a mean diameter of 30 ± 2 nm (ferritin oligomers) and the second elution peak with a monodisperse diameter of 10 ± 1 nm (monomeric ferritin) [46].

The chromatographic profile of the FTH-AuNP sample (A_280nm_) shows elution peaks at 8.5 and 11.2 mL, while the FTL-AuNP sample shows protein elution peaks at 8.6 and 11.4 mL (Figure 3B,C, respectively). The absorbance profile of these samples at 550 nm shows the coelution of AuNPs with the first protein elution peak (8.5–8.6 mL), which corresponds to the fraction containing oligomeric ferritin species. On the other hand, the lack of absorption at A_550nm_ in the ferritin monomer elution (second elution peak, 11.2–11.4 mL) suggests that this peak contains apoferritin or ferritin containing gold nanoclusters, which are fluorescent and are undetectable by absorbance measurements [15,48,49].

A semiquantitative analysis of the peak areas from protein chromatograms in Figure 3 suggests that nanoparticle synthesis in ferritin induces changes that alter the monomer/oligomer ratio present in the initial apoferritin sample (Table 2). This monomer/oligomer ratio is lower for FTH-AuNP (ratio: 1.8), which implies a higher proportion of oligomeric species (fraction associated with AuNP) and, therefore, a higher AuNP synthesis efficiency compared to FTL-AuNP. The FTH homopolymer has a ferroxidase center in each of its 24 subunits that could interact through the imidazole ring of His residues with Au^+3^ ions [17,50], offering nucleation sites for the further formation of nanostructures. In addition, sulfur-containing amino acids (cysteine and methionine) in the FTH homopolymer could play an essential role in interacting with Au^+3^ ions [15,30,51]. Nanoparticle nucleation and growth in FTL seem to be mediated by Cys126 [28].

The measurement of the hydrodynamic diameter (H_d_) by dynamic light scattering (DLS) of the apoferritin samples displayed an average size near 20 nm, higher than the size of monomeric ferritin (12 nm), confirming the presence of monomeric and oligomeric protein species in the starting material (Table 3). Furthermore, in ferritin-AuNP samples, H_d_ values increased, thus reconfirming that AuNP synthesis induces oligomeric protein species, consistent with the results obtained by SEC analysis. Ferritin oligomer formation related to AuNP synthesis was also observed by native PAGE analysis (Figures S1 and S2).

The net electrostatic charge of folded apoferritin (Z-potential) in Tris-HCl buffer at pH 7 was measured for both types of apo-homopolymer samples, showing a result of nearly −13 mV (Table 3), representing an overall view of ferritin surface potentials, including contributions from solvent and tightly bound metal. After AuNP synthesis, the Z-potential in both homopolymers decreased to −10.2 and −7.6 mV for FTH-AuNP and FTL-AuNP, respectively. The net charge decrease is caused by the interaction between the protein with Au ions, and the lowered electrostatic repulsion between ferritin monomers can trigger the formation of ferritin oligomers. Generally, Z-potential values are not reported in studies involving metallic nanoparticle synthesis in ferritin, making it difficult to compare our results with others. Exceptionally, previous studies showed a decrease in Z-potential and increased H_d_ in iron-loaded ferritin compared to apoferritin [46,52,53].

Welch et al. demonstrated that ferritin containing H-chains (FTH) is oxidized during iron loading at its ferroxidase center, and this oxidation results in protein aggregation. The cysteine at position 90, located at the end of the solvent-facing loop, is critical for ferritin aggregate formation during iron loading. Additionally, dityrosine moieties are formed during iron loading, depending on cysteine residue oxidation [54,55]. Our results show that both FTH and FTL form oligomers, suggesting a possible role of the process of AuNP synthesis that could indirectly lead to the oxidation of surface-exposed cysteines.

Another suitable explanation for ferritin oligomerization after AuNP synthesis is that gold interacts with the side chains of certain amino acids, which could potentially induce small changes in the secondary structure of the protein that could affect the external surface of ferritin, somehow decreasing the repulsion between protein nanocages mediated by intermolecular interactions. So far, side-chain conformational changes in the amino acid that interacts with Au^+3^ or Au^0^ have been reported [28,51]. In addition, different authors have reported the conservation of the secondary structures of ferritin after iron incorporation to produce magnetoferritin, therefore maintaining the almost intact protein cage after iron mineralization [56,57,58,59]. Identical results were reported by Xiangyou Lui et al., revealing that ferritin with Pt nanoparticles does not have significant changes in the secondary structures of the protein, markedly α-helical [60]. On the contrary, Kashanian et al. [61] reported that the synthesis of cobalt nanoparticles inside apoferritin induces an increment of near 9% in the α-helical content and a decrease in the β-sheet protein structure content, thus altering colloidal stability. Therefore, there is not enough evidence supporting that the formation of oligomeric species after the synthesis of inorganic nanoparticles in ferritin is related to the conformational changes of the secondary structure.

**Figure 4:**
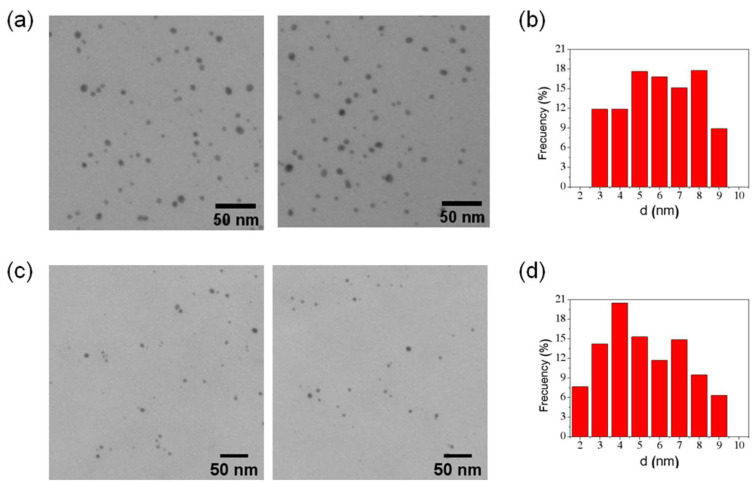
TEM images of FT-AuNP samples without staining. (**a**) FTH-AuNP, (**b**) size distribution of FTH-AuNP samples, (**c**) FTL-AuNP and (**d**) size distribution of FTL-AuNP samples. Scale bar 200 nm.

We studied FT-AuNP samples using TEM to measure the gold nanoparticle size distribution of each ferritin homopolymer. AuNPs were directly observed, allowing the measurement of the electron-dense spherical metallic cores, showing that the mean diameter of the nanoparticles was 6 ± 1.8 nm (N = 833) and 5 ± 1.9 nm (N = 444) nm for FTH-AuNP and FTL-AuNP, respectively (Figure 4). Although these results cannot directly demonstrate that AuNPs are inside ferritin’s cavity, the absence of AuNPs with sizes over 9 nm is evidence that AuNPs could be inside the protein, given that the ferritin cavity has a ~8 nm size constraint. Additionally, the negative-stained samples of FTH-AuNP (Figure S3) showed some electron-dense cores inside the ferritin structure, which had an unchanged mean diameter of 11.5 ± 0.8 nm (N = 100). No electron-dense cores are observed outside the protein-stained shell. Nevertheless, the presence of Au atoms on the ferritin surface, driving the formation of the observed oligomeric species, cannot be excluded.

### 3.2. Cell Viability and FT-AuNP Toxicity

**Figure 5:**
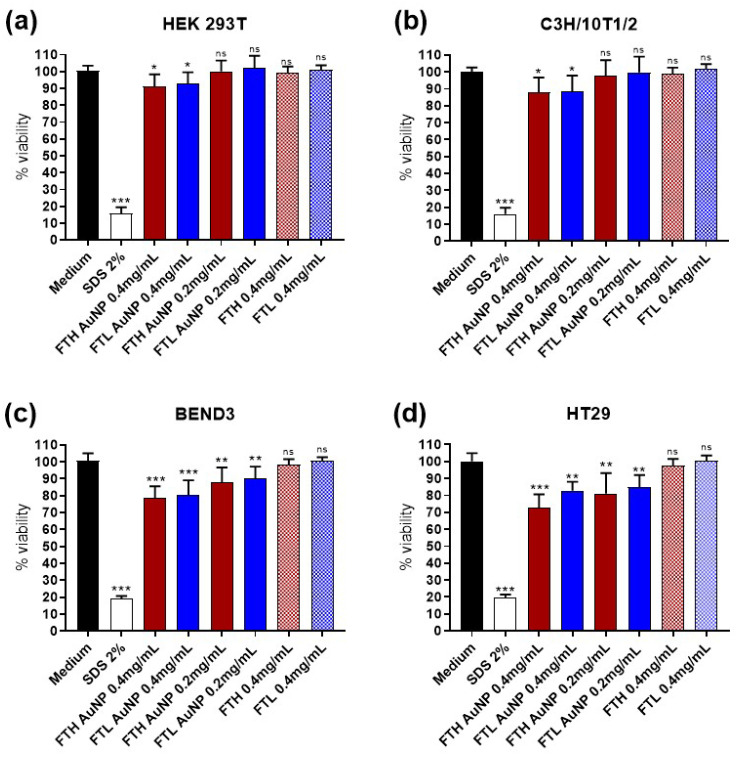
Cell viability after incubation with ferritin Au-NP compared to apoferritin. (**a**) HEK 293T cell line, (**b**) C3H/10T1/2 cell lines, (**c**) BEND3 cell lines, and (**d**) HT29 cell lines (mean ± SEM; *n* = 9; 3 independent experiments with 3 replications each); MTS assay. Data were analyzed using a one-way analysis of variance and Tukey’s post hoc test. Statistically significant differences are indicated (* *p* < 0.05; ** *p* < 0.01; *** *p* < 0.001; ns, not significant).

We used a proliferation assay to evaluate cell viability after 24-h incubation with FTH and FTL containing AuNPs. Results indicated that the incubation with the highest concentration of 0.4 mg/mL (0.8 μM) of FT-AuNPs had a minor effect on the viability of human HEK293T cells and murine C3H/10T1/2 cells, considered as healthy control cells (Figure 5). Two other cell lines were tested for viability after incubation with FT-AuNP, colon cancerous HT29 cells, and neural endothelial BEND3 cells. Both showed more sensitivity to the treatment, triggering a reduction of cell viability even at 0.2 mg/mL of FT-AuNP concentration. However, the viability of these two cell lines after doubling the amount of FT-AuNP never fell below 70%. The increased toxicity observed in the HT29 and BEND3 lines may be caused by a higher level of internalization, showing accumulation or dose-dependent behavior, as defined by other authors [16,62].

A broad spectrum of toxicities reported for AuNP depends on many factors: shape, size, capping agent, the cell line used in the viability assay, and exposition time, among others [63]. In this study, using ferritin for AuNP capping decreases reactivity, reduces toxicity, and offers a biocompatible carrier. The detected relative toxicity found for FTH-AuNP and FTL-AuNP could be considered low when compared to other values reported for AuNP in gold-containing ferritin, which range between 100% to 90% viability in SH-SY5Y cells (72 h incubation, up to 30 μM [29]), Caco-2, and HepG2 cells (12 h incubation, up to 10 μM [16]).

### 3.3. Cell Uptake of FTH and FTL with Gold Nanoparticles

**Figure 6:**
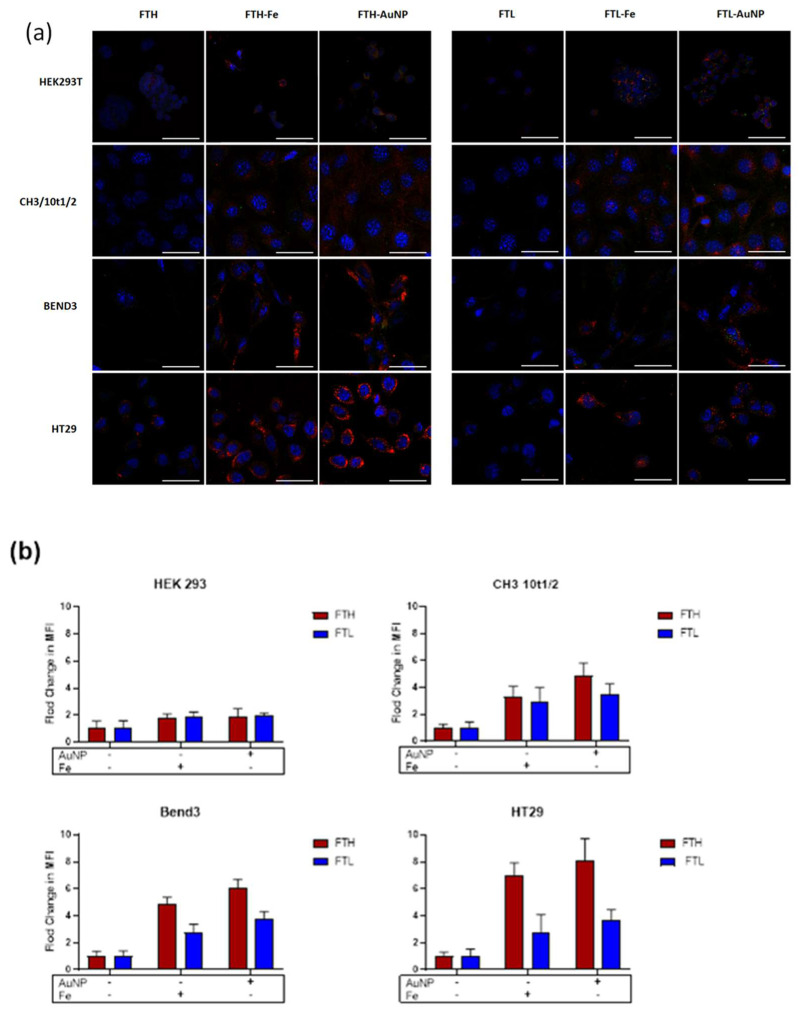
Confocal microscopy results for different cell lines after incubation with FT-AuNP. (**a**) Confocal images of HEK293T, CH3/10T1/2, BEND3, and HT29 were obtained after incubation for 24 h with H-ferritin (FTH) and L-ferritin (FTL), with or without AuNP or iron oxides (anti FTH or FTL in red; (DAPI) nucleus in blue; scale bars: 20 μm). (**b**) Plots representing fold changes in MFI (mean fluorescence intensity) ± SD from more than three independent experiments.

The uptake of FT-AuNP by different cell lines was analyzed after 2 h of incubation by confocal microscopy (Figure 6A). Qualitatively, the uptake of FTH or FTL containing AuNPs was higher compared to apoferritin. This uptake enhancement was also observed for ferritin containing iron oxides (FT-Fe), used as control samples (red channel in Figure 6A). Image quantification corroborated the differences in cell internalization of the ferritin samples, which were consistently higher for ferritins containing AuNPs or iron oxides (Figure 5B). For the HEK 293T cell line, the internalization values varied between 1.1- to 1.8-fold more than apoferritin and were similar for both homopolymers and metallic contents (FT-AuNP or FT-Fe). For the C3H/10T1/2 cell line, the FTH homopolymer containing AuNP and Fe showed higher internalization values (between 2.4 and 2.0, respectively) than FTL-AuNP and FTL-Fe (1.9 and 1.6, respectively). In BEND3 and HT29 cell lines, FTH-AuNP was highly internalized, reaching values of 4.9- and 6.4-fold more than FTH, respectively. FTH-Fe also showed increased internalization rates, with values of 3.3- and 4.7-fold more than FTH in BEND3 and HT29, respectively. In these two cell lines, FTL containing AuNP or iron oxides showed a lower uptake increase than FTH, with a 2.8- and 2.2-fold increase over apo-FTL, respectively (Figure 6B).

The differences in uptake between both homopolymers, FTH and FTL, containing either AuNP or iron oxides, can be attributed to the availability of their uptake receptors. H-chains are mainly internalized by transferrin receptor 1 (TR1), which is ubiquitously expressed and overexpressed in cancerous and vascular endothelial cell lines [64]. On the other hand, L-chains are internalized by the SCARA5 receptor, which is less abundant in cells and only expressed in particular cell types [39,65]. Our results are in congruence with previous TfR1 expression data for the HEK 293T cell-line, which shows low levels of internalization compared with other cell lines and has been described to have low levels of TfR1 [31,66]. Additionally, the high internalization observed in the BEND3 and HT29 cell lines correlates with the previously reported overexpression of TfR1 in these cell lines [67,68]. These results are relevant for developing new imaging or drug-delivery systems based on gold-containing ferritin for brain targeting and cancer treatments where cells overexpress transferrin receptors.

Notoriously, metallic content also affects the uptake level of both homopolymers. This observation is best appreciated in cell lines with enhanced uptake, such as BEND3 and HT29, suggesting that apoFTH and apoFTL are poorly internalized by these cell types; however, the same homopolymers with a metallic content are highly internalized, although in a much lower level for FTL. The same behavior has been previously observed by Sunkesula et al. [69], who observed that ferritin iron content modulates its uptake by the intestinal epithelium. However, this is the first time this observation has been made for ferritin with other contents, particularly AuNPs.

Cell internalization of ferritin occurs through receptor-mediated endocytosis (RME), which provides one major pathway for the trafficking of extracellular molecules into the cell. The first step of RME involves the binding of a ligand to a specific cell surface receptor. Circulating ferritin needs to overcome this first step to enter the cell successfully. Thus, the question arising from this investigation is whether there is a difference in receptor binding between apoferritin and holoferritin, in this case, gold-containing ferritin.

Studies comparing apo and holoprotein structures show that, in most cases, the proteins undergo relatively small conformational rearrangements of their tertiary structure upon ligand binding/release (root mean square deviations (RMSDs) of the a-carbon from the native are <1 A°) [70]. Clark et al. confirmed an inherently different behavior for backbones and side chains, where backbones tend to show very little conformational change, and side chains are frequently pushed into new conformations upon ligand binding [71]. Additionally, molecular dynamics of apo and holo-proteins indicated that, although the overall protein structure is unchanged by the presence of the ligand, other interaction properties are significantly affected by ligand binding, such as bound waters. This antecedent suggests that binding thermodynamics depend not simply on ligand interactions with a small subset of protein atoms but dynamically on the range of motions coupling water, protein, and ligand molecules [72,73].

Future work should be directed towards a better understanding of how metallic content interacts with ferritin to induce an enhancement in its cellular uptake.

## 4. Conclusions

When comparing the two human ferritin homopolymers, FTH and FTL, our results demonstrate that AuNP can be synthesized in both, but nanoparticle synthesis in FTH gives slightly bigger nanoparticles and is more efficient than FTL. In addition, FTH-AuNP has a higher degree of cellular uptake in three of the cell lines tested compared to FTL-AuNP, which correlates with previously reported TfR1 expression in these lines. Inorganic content (AuNP or iron oxides) enhances both homopolymers’ cellular internalization, especially in FTH, and AuNP has a higher effect on internalization rates than iron oxides in our study. In summary, we show that cellular ferritin uptake seems to depend on three independent factors: (a) the protein chain forming the ferritin cage (and probably the ratio between chains for heteropolymeric ferritins), (b) the presence or absence of metallic cargo within ferritin, and (c) the presence and abundance of specific receptors on the cell membrane. The highest ferritin uptake was achieved using AuNP-loaded heavy-chain ferritin homopolymers in transferrin-receptor-rich cell lines, while apoferritin is always poorly internalized.

## Figures and Tables

**Table 1:** Sequences of primers used in this work with the introduced restriction endonuclease sites.

Primer Name	Sequence 5′–3′
FTH-F	TCGCCCATATGACGACCGCGTCCACCTC
FTH-R	TTGGTCTCGAGGGAAGTCACCCCACGGCTATG
FTL-F	AGCCACATATGAGCTCCCAGATTCGTCAG
FTL-R	GGGCCCTCGAGAAGTCGCTGGGCTCAGAAG

**Table 2:** Monomeric/oligomeric fraction analysis from size exclusion chromatograms.

	Apo FTL	FTL-AuNP	Apo FTH	FTH-AuNP
Monomer	60.1%	74.4%	73.3%	64.3%
Oligomer	39.9%	25.6%	26.7%	35.7%
Ratio	1.5:1	2.9:1	2.7:1	1.8:1

**Table 3:** Monomeric/oligomeric fraction analysis from size exclusion chromatograms.

Sample	H_d_ (nm)	Z-Pot (mV)
Apo FTH	18 ± 12	−13.5
Apo FTL	20 ± 9	−13.7
FTH-AuNP	55 ± 29	−10.2
FTL-AuNP	35 ± 16	−7.6

## Data Availability

The data presented in this study are available on request from the corresponding author.

## Supplementary Materials

The following are available online at https://www.mdpi.com/article/10.3390/pharmaceutics13111966/s1, Figure S1: Native PAGE of ferritin samples, Figure S2: FTH-AuNP SEC chromatogram (a) and Native PAGE of elution fractions (b), Figure S3: TEM analysis for FTH-AuNP samples with negative staining.
