# Supplementary material for: Enhanced Cellular Uptake of H-Chain Human Ferritin Containing Gold Nanoparticles
Source: Pharmaceutics. 2021 Nov 19;13(11):1966. doi: 10.3390/pharmaceutics13111966 (PMC8623468; doi:10.3390/pharmaceutics13111966)
Supplement: Supplementary file 1 [file pharmaceutics-13-01966-s001.zip › pharmaceutics-1426818-supplementary.pdf]

pharmaceutics

# Supplementary Materials: Enhanced Cellular Uptake of H-Chain Human Ferritin Containing Gold Nanoparticles

Italo Moglia, Margarita Santiago, Simón Guerrero, Mónica Soler, Álvaro Olivera-Nappa and Marcelo J. Kogan

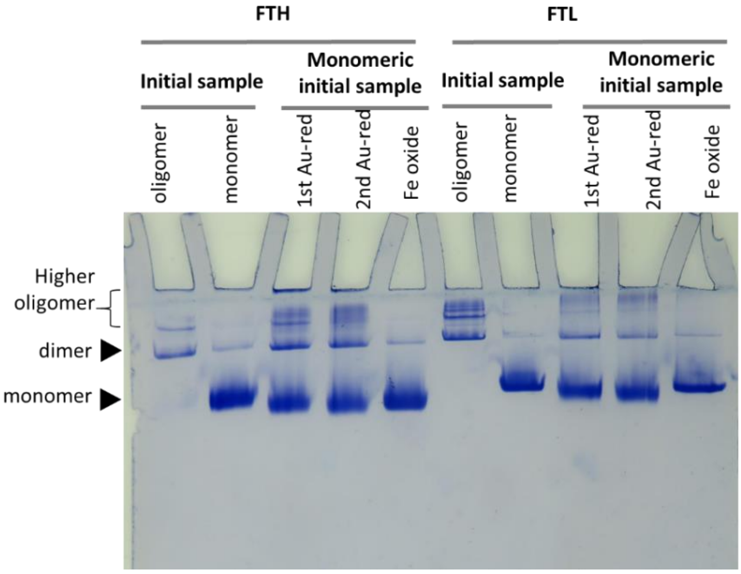


**Figure S1.** Native PAGE of ferritin samples.

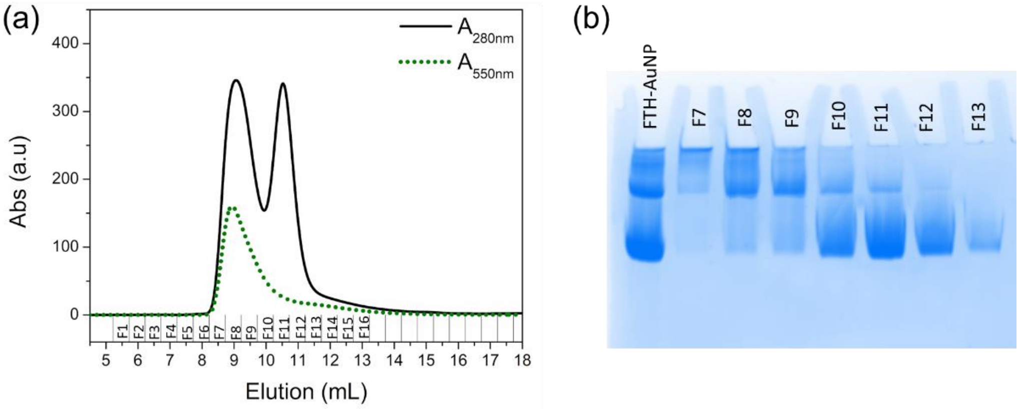


**Figure S2.** FTH-AuNP SEC chromatogram (**a**) and Native PAGE of elution fractions (**b**).

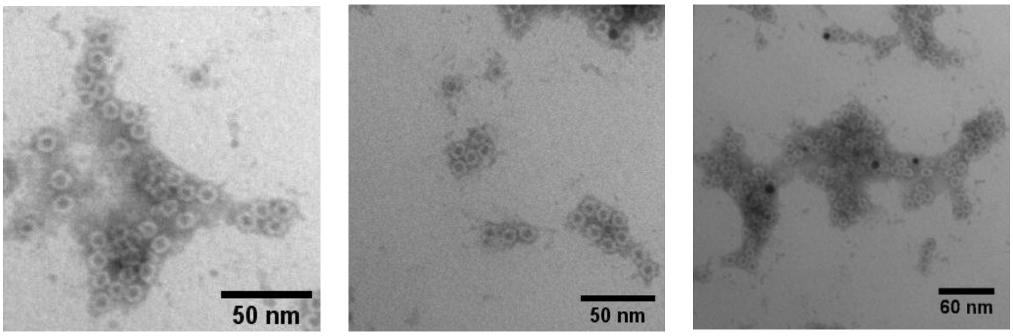


**Figure S3.** TEM analysis for FTH-AuNP samples with negative staining.
